# Temporal stability of parent-reported behavior problems in late talkers over 2 years: a prospective case-control study from toddlerhood to preschool age

**DOI:** 10.1186/s11689-022-09445-x

**Published:** 2022-06-17

**Authors:** Hsin-Hui Lu, Jeng-Dau Tsai, Feng-Ming Tsao

**Affiliations:** 1 Department of Psychology, Chung Shan Medical University, Taichung, Taiwan; 2 Clinical Psychological Room, Chung Shan Medical University Hospital, Taichung, Taiwan; 3 Department of Pediatrics, Chung Shan Medical University Hospital, Taichung, Taiwan; 4 School of Medicine, Chung Shan Medical University, Taichung, Taiwan; 5 Department of Psychology and Imaging Center for Integrated Body, Mind and Culture Research, National Taiwan University, Taipei, Taiwan

**Keywords:** Early identification, Behavior problems, Late talkers, Temporal stability

## Abstract

**Background:**

Late talking (LT) in toddlers is a risk factor for language weakness that may interfere with the development of using language to regulate behavior and emotion and contribute to the development of behavior problems from early childhood. This study examined the temporal stability of parent-reported behavior problems among Mandarin-speaking LT toddlers from ages 2 to 4 in Taiwan.

**Methods:**

Thirty-one LT and 31 typical language development (TLD) toddlers were assessed for their vocabulary production at age 2 with the Words and Sentences Forms of the MacArthur-Bates Communicative Developmental Inventories Toddler Form (Taiwan version). Additionally, participants’ receptive and expressive language abilities were assessed using the receptive and expressive language subscales of the Bayley Scales of Infant and Toddler Development, Third Edition. At age 4, the Child Language Disorder Scale-Revised was applied and included the two core subtests for auditory comprehension and expressive communication. At ages 2 and 4 years, behavior problems were assessed with the Child Behavior Checklist.

**Results:**

There was a higher percentage of participants with persistent behavior problems among LT toddlers than among TLD toddlers. Moreover, toddlers with larger vocabularies were less likely to develop withdrawal behaviors by preschool age.

**Conclusions:**

This study supported the temporal stability of parent-reported behavior problems among LT toddlers across early childhood. Early identification of and intervention for behavior problems associated with LT in toddlerhood is essential to alleviate their behavior problems later in preschool years.

## Introduction

The term *late talkers*, i.e., late-talking (LT) toddlers, refers to children aged 18–35 months who experience delayed onset of language but have no other diagnosed disabilities or developmental delays in neurological, sensory, cognitive, or motor domains [1, 2]. LT toddlers represent approximately 10–15% of the toddler population [3, 4]. There is extensive evidence that LT is a risk factor for language weakness persisting into adolescence [5]. Language delays may interfere with the development of using (self-directed) language to regulate behaviors [6] and emotion [7, 8] and may contribute to the development of behavior problems from early childhood.

Multiple panel studies have reported behavior problems among late talkers in toddlerhood [9–13], and late talking has been shown to be a predictor for such problems in childhood and adulthood [3, 14–17]. Internalizing and externalizing behaviors may follow different developmental pathways [18]. Previous studies found that a 2-year-old children with low receptive language abilities were at risk of internalizing behaviors [19, 20]. However, whether LT toddlers with behavior problems in later developmental stages (e.g., preschool age) show temporal stability from early behavior problems was unclear.

In fact, there has been very little panel research examining whether LT toddlers with behavior problems in later developmental stages (e.g., preschool age) show temporal stability from early behavior problems. Temporal stability—also known as developmental continuity—of behavior problems refers to a persistent presence of behavior problems over time. Temporal stability of early behavior problems has been found in typical language development (TLD) children; furthermore, temporal stability of behavior problems emerging in early life may be a predictor for the emergence of psychopathology [21, 22]. If the behavior problems of LT toddlers were stable across early childhood, early identification of and intervention for LT toddlers with behavior problems would be useful in reducing their risk of mental health problems later in life [23, 24].

This 2-year prospective case-control study examined the temporal stability of behavior problems from toddlerhood to preschool age in a community sample of LT and TLD toddlers. Based on developmental continuity and persistent poor language development in LT toddlers, we hypothesized that the percentage of LT toddlers with persistent behavior problems through early childhood would be higher than that of TLD toddlers with ongoing behavior problems. Moreover, we examined whether toddlers with larger vocabularies were less likely to have concurrent behavior problems (i.e., in toddlerhood) and develop them over time (i.e., by preschool age). These findings will deepen understanding of the associations between early language delay and temporal stability of behavior problems across various early developmental stages.

## Methods

### Participants

**Fig. 1:**
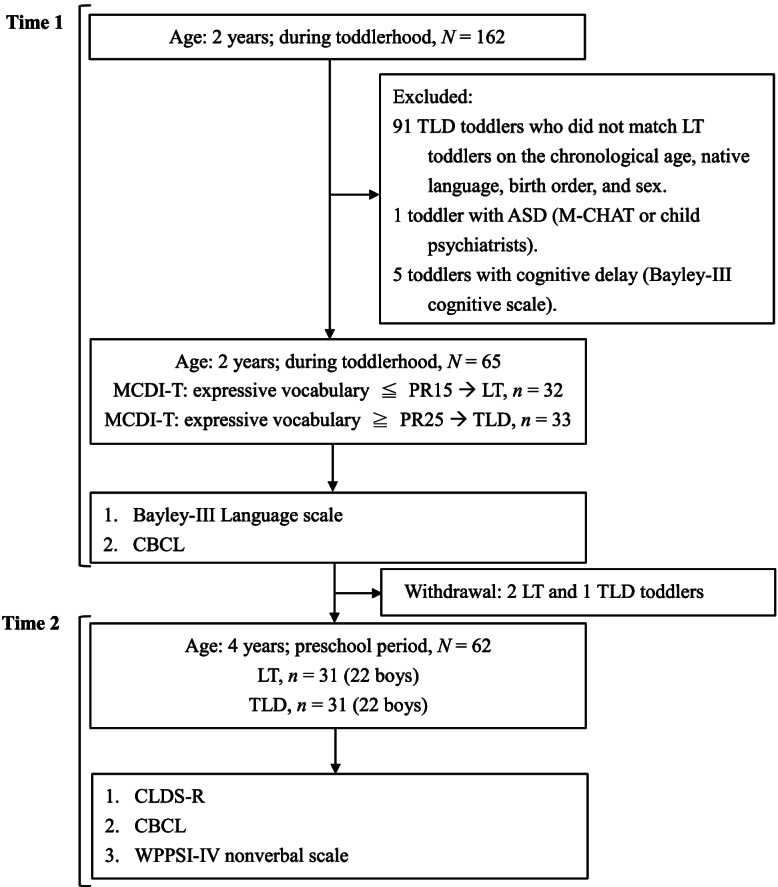
Flowchart of study design, *N* = 62. Notes: *ASD*, autism spectrum disorders; *CLDS-R*, Child Language Disorder Scale-Revised; *MCDI-T*, Mandarin-Chinese version of the MacArthur-Bates Communicative Developmental Inventories Toddler; *M-CHAT*, Modified Checklist for Autism in Toddlers

This prospective case-control cohort study comprised two waves of data collection. A total of 162 24–33-month-old toddlers were recruited from parenting websites or local pediatric clinics in northern Taiwan (Fig. 1). We assessed their expressive vocabulary using the Mandarin Chinese version of the Words and Sentences Forms of the MacArthur-Bates Communicative Developmental Inventories Toddler Form (MCDI-T) [25] to screen toddlers with delayed expressive language. Out of 162 toddlers, 65 were included in the data collection at time 1. The matching criteria for LT toddlers included chronological age (within 1 month of birthdate), native language (Mandarin), birth order, and sex. Due to the strict matching criteria, 91 TLD participants who did not match LT participants on the criteria mentioned above and who completed only the MCDI-T were excluded from the final data analysis. Furthermore, one toddler diagnosed with autism spectrum disorder (ASD) and five toddlers with cognitive delays were also excluded. Among the 65 participants who completed both waves of data collection, 32 toddlers were in the LT group, and 33 were in the TLD group. The inclusion criteria for LT toddlers were the same as those in other studies [15, 26–28]. In particular, for the LT group, word production performance should be at or below the 15th percentile on the MCDI-T, whereas that for the TLD toddlers should be at or above the 25th percentile. Behavior problems of children were collected via parental reports [29]. One LT toddler and two TLD toddlers did not participate in subsequent follow-up (time 2) and were excluded. For the final data analysis of this longitudinal study, 31 LT toddlers (22 boys) and 31 TLD toddlers (22 boys) completed the data collection. The mean TLD and LT participant ages for the two data collection points were 27.59 (*SD* = 2.46) and 27.54 (*SD* = 2.60) months at time 1 and 50.88 (*SD* = 2.46) and 51.37 (*SD* = 2.53) months at time 2, respectively.

At time 1, the Modified Checklist for Autism in Toddlers (M-CHAT) [30] was used as a screening tool to identify toddlers at high risk for ASD, and the Bayley Scales of Infant and Toddler Development, Third Edition (Bayley-III) [31] was used to identify children with cognitive delays. Furthermore, according to parental reports, all toddlers were born full term (gestational age > 36 weeks) with birth weights over 2500 g; no complications were encountered during pregnancy or delivery. Additionally, the participants had no other critical incidents, chronic diseases, or sensory-motor deficits.

Table 1 presents the participants’ demographic characteristics at age 2 (time 1). Although there were no differences between the TLD and LT groups (*p*s > .05) in demographic factors, 16.13% of TLD toddlers had a history of otitis media compared to 9.68% of LT toddlers. We also observed that few TLD or LT toddlers attended daycare. Approximately, 50% of TLD and LT toddlers had their parents as their primary caregivers. Over 70% of TLD and LT toddlers’ parents were educated at the university level and above, and over 50% of the toddlers were from middle- or high-income families. The two groups were similar in the history of otitis media, language exposure environment, and socioeconomic status.

**Table 1:** Sociodemographic characteristics of participants at age 2

Variables	TLD	LT	*p*
Sex			1.000
Male	22 (70.97)	22 (70.97)	
Female	9 (29.03)	9 (29.03)	
The history of otitis media	5 (16.13)	3 (9.68)	.452
Attending daycare	3 (9.68)	5 (16.13)	.257
Caregiver, day/night			.617
Parent/parent	18 (58.06)	15 (48.39)	
Grandparent/parent	5 (16.13)	8 (25.81)	
Nanny/parent	8 (25.81)	8 (25.81)	
Mother educational level			.195
Senior high school	1 (3.23)	5 (16.13)	
University and above	30 (96.77)	26 (83.87)	
Father educational level			.182
Junior high school	0 (0.00)	1 (3.23)	
Senior high school	3 (9.68)	7 (22.58)	
University and above	28 (90.32)	23 (74.19)	
Annual family income (NTD^a^)			.410
< 650,000	5 (16.13)	7 (22.58)	
650,000–1,000,000	7 (22.58)	9 (29.03)	
> 1,000,000	19 (61.69)	15 (48.39)	

*TLD* typical language development (*n* = 31), *LT* late talking (*n* = 31); data are presented as *n* (%). *p* derived from chi-square tests and Fisher’s exact test (cell cases < 5). ^a^NTD, Taiwan dollar

Table 2 presents participants’ cognitive and language scores at ages 2 (time 1) and 4 (time 2). No significant differences were noted in cognitive abilities by age in either of the two groups (*p*s > .05). The LT group exhibited lower receptive and expressive language skills than the TLD group at ages 2 [*F*s(1, 60) = 16.48 and 143.65, respectively, *p*s < .001, *η*_p_^*2*^s = .22 and .71] and 4 [*Fs*(1, 60) = 53.68 and 70.63, *p*s = .001, *η*_p_^*2*^s = .47 and .54].

**Table 2:** Cognitive and language scores in the TLD and LT groups

Scores	TLD	LT	*p*
Time 1 (age 2)			
Cognition^a^	109.52 (10.03)	105.16 (12.88)	.143
Receptive^b^	0.54 (0.58)	−0.06 (0.59)	< .001
Expressive^b^	−0.09 (0.45)	−1.35 (0.38)	< .001
Time 2 (age 4)			
Cognition^c^	102.06 (9.61)	98.71 (8.51)	.131
Receptive^d^	0.56 (0.39)	−0.72 (0.89)	.001
Expressive^d^	−0.22 (0.41)	−1.48 (0.74)	.001

*TLD* typical language development group (*n* = 31); *LT*, late-talking group (*n* = 31). Data are presented as mean (SD). *p*, derived from ANOVA. ^a^Bayley-III Cognitive Scale Score (100 ± 16). ^b^Bayley-III Language Scale (z scores). ^c^WPPSI-IV (Mandarin-Chinese version): index of nonverbal cognition (100 ± 15). ^d^CLDS-R (z scores)

### Measures

#### Assessment of language abilities

We assessed the participants’ vocabulary production at age 2 with the Words and Sentences Forms of the MCDI-T [25], which has been used to assess toddlers aged 16–36 months. Additionally, the participants’ receptive and expressive language abilities at age 2 were assessed using the receptive and expressive language subscales of the Bayley-III, which has been used to assess toddlers aged 16–42 months [31].

The Mandarin Chinese version of the Child Language Disorder Scale-Revised (CLDS-R) [32] was employed to assess participants at age 4 and included the two core subtests for auditory comprehension and expressive communication. The CLDS-R has been used to assess children aged 3–6 years.

#### Assessment of behavior problems

At ages 2 and 4 years, behavior problems were assessed using the Mandarin Chinese version of the Child Behavior Checklist (CBCL-MC) for children aged 1.5 to 5 years [33]. Parents rated each item as 0 (*not true*), 1 (*somewhat or sometimes true*), or 2 (*very true or often true*). The scores were aggregated on eight syndrome subscales and three main scales. Scores on the emotionally reactive (e.g., “Disturbed by any change in routine”), anxious/depressed (e.g., “Unhappy, sad, or depressed”), somatic complaints (e.g., “Headaches (without medical cause)”), and withdrawn (e.g., “Shows little affection toward people”) subscales were aggregated to comprise the internalizing scale score. Similarly, the attention (e.g., “Can’t sit still, restless, or hyperactive”) and aggression (e.g., “Demands must be met immediately”) subscale scores were aggregated to comprise the externalizing scale score. Finally, the remaining two subscales, sleep problems (e.g., “Wakes up often at night”) and other problems, were combined with the internalizing and externalizing scales to comprise the total problems scale score. For the three main scales and seven syndrome subscales, except for the other problems syndrome subscale, raw scores could be converted to normalized T scores. The behavior problems status for the individual main scale and subscale scores were dichotomized as elevated (1 = *the presence of behavior problems*) or normal (0 = *the absence of behavior problems*). According to the CBCL’s definition, subscale scores below the 93rd percentile are considered normal, whereas scores at or above the 93rd percentile (T score ≥ 65) are considered elevated. For the major scales, scores below the 85th percentile are considered normal, whereas scores at or above the 85th percentile (T score ≥ 60) are considered elevated.

#### Assessment of cognitive abilities

The cognitive scale of the Bayley-III [31], administered in Mandarin Chinese, was employed to evaluate the participants’ cognitive abilities at age 2. This scale has been applied to children from birth to 42 months. At age 4 (i.e., over 42 months old), the participants’ cognitive abilities were measured using the Mandarin Chinese version of the Nonverbal Index (NVI) of the Wechsler Preschool and Primary Scale of Intelligence, Fourth Edition (WPPSI-IV) [34]. This test was designed for assessments in children aged 2 years and 6 months to 7 years and 11 months.

### Procedure

All tests were administered in a quiet room. Before testing, the researcher informed the parents of the research procedures, after which the parents provided their informed consent. The test administration duration for each wave was 1–1.5 h. Furthermore, the Bayley-III, the WPPSI-IV, and the CLDS-R tests were administered by a licensed clinical psychologist specializing in developmental psychopathology. The Research Ethics Committee of National Taiwan University, Taiwan, approved this study.

### Data analysis plan

The aim of the study was to examine the temporal stability of behavior problems in LT children compared to TLD children from toddlerhood to preschool age. First, to understand the relationship between changes in T scores from ages 2 to 4 in the LT and TLD groups and total problems, a group (LT versus TLD) by time (age 2/time 1 versus age 4/time 2) mixed-model ANOVA was performed. To assess the relationship between changes in T scores at both ages in the two groups and internalizing and externalizing behaviors and syndromes, 2 × 2 repeated-measures multivariate analyses of variance (MANOVA) was performed to adjust for chance significance due to multiple testing. Additionally, sleep problems subscale scores were not included in the internalizing and externalizing scales; rather, a 2 × 2 mixed-model ANOVA was conducted to test the sleep problem syndrome scores. The above tests adopted a significance level (α) of *p* < .05. Furthermore, each MANOVA was followed by 2 × 2 mixed-model ANOVAs for each dependent variable; where appropriate, degrees of freedom were adjusted using the Greenhouse-Geisser procedure when indicated necessary by Mauchly’s sphericity test. Furthermore, *p*-values were adjusted according to the Bonferroni procedure to guard against type-1 errors. Therefore, follow-up ANOVAs with internalizing and externalizing behaviors as dependent variables adopted a significance level (α) of *p* < .025 (=.05/2), those with internalizing syndromes (emotionally reactive, anxious/depressed, somatic complaints, and withdrawn) as dependent variables adopted *p* < .0125 (=.05/4), and those with externalizing syndromes (attention and aggression) as dependent variables adopted *p* < .025 (=.05/2). Effect sizes from the ANOVAs and MANOVAs were calculated using partial eta square (*η*_p_^2^), which can be translated directly into percent of variance explained. Cohen [35] provided a basic framework for interpreting these effects as small (*η*_p_^2^ = .01), moderate (*η*_p_^2^ = .06), or large (*η*_p_^2^ = .15). Due to small sample sizes, *p*-values less than or equal to 0.10 and *η*_p_^2^ greater than or equal to .06 were considered to indicate trends. If behavior problems of LT children were more temporally stable than those of TLD children during toddlerhood and preschool age, a significant main effect of group would be found in the above tests.

Next, Fisher’s exact tests were conducted to test the differences in the percentages of participants with behavior problems between the LT and TLD groups for each of the CBCL scales and subscales at ages 2 and 4. Fisher’s exact test was also used to further understand whether the proportion of the presence of behavior problems at ages 2 and age 4 versus only at age 2 differed between LT children and TLD children. These tests were considered significant using a Bonferroni-corrected α level, as in the ANOVAs mentioned above. Due to small sample sizes, *p*-values less than or equal to 0.10 were considered to indicate trends. Finally, with each CBCL scale and subscale, the association of vocabulary size with concurrent behavior problems (i.e., at age 2) and those that develop over time (i.e., at age 4) among toddlers was examined by logistic regression analyses. All statistical analyses in this study were conducted using IBM SPSS Statistics 22.0.

## Results

### Behavior problem T scores: MANOVAs and ANOVAs

Table 3 shows the T scores of the ten CBCL scales and subscales for the LT and TLD groups at ages 2 and 4. The association was assessed between T-score changes from age 2 to 4 in the LT and TLD groups and each of the CBCL scales and subscales.

**Table 3:** T scores for CBCL scales and subscales in the TLD and LT groups

	Time 1 (age 2)				Time 2 (age 4)									
	TLD		LT		TLD		LT		*F*(1, 60)_(*G*)_		*F*(1, 60)_(*T*)_		*F* (1, 60) _(G × T)_	
	Mean	SD	Mean	SD	Mean	SD	Mean	SD	*p*^a^	*η*_p_^2^	*p*^a^	*η*_p_^2^	*p*^a^	*η*_p_^2^
Total problems	51.48	8.83	55.74	9.46	51.19	8.17	55.10	9.90	.046*	.07	.688	.00	.879	.00
Internalizing problems	51.42	8.88	54.13	10.25	53.71	8.55	56.64	10.86	.208	.03	.027^**+**^	.08	.916	.00
Externalizing problems	48.81	9.18	51.55	8.25	46.71	8.14	49.74	7.90	.099	.05	.124	.04	.908	.00
Emotionally reactive	55.48	5.79	57.68	6.34	54.87	6.08	58.62	8.18	.036^**+**^	.07	.869	.00	.429	.01
Anxious/depressed	53.23	3.94	55.94	6.06	55.52	5.05	58.87	9.36	.030^**+**^	.08	.005*	.13	.719	.00
Somatic complaints	55.42	4.48	54.97	5.75	56.81	6.17	56.55	6.99	.763	.00	.120	.04	.918	.00
Withdrawn	55.81	5.37	58.94	9.71	55.94	6.17	58.61	8.14	.077	.05	.926	.00	.827	.00
Attention	53.23	4.54	53.81	5.74	52.61	3.89	53.23	3.40	.504	.01	.404	.01	.982	.00
Aggression	52.94	4.78	54.65	5.71	52.45	4.63	53.42	4.96	.179	.03	.300	.02	.652	.00
Sleep problems	56.94	5.48	59.35	9.68	56.00	5.14	58.42	7.91	.125	.04	.355	.01	1.000	.00

*TLD* typical language development group (*n* = 31); *LT*, late-talking group (*n* = 31), *SD* standard deviation, *T* time, *G* group. *p* and *η*_p_^2^ derived from one-way 2 × 2 ANOVA. ^a^Based on Bonferroni-corrected α level and *η*_p_^2^, *significant, ^**+**^borderline significant

#### Main problems (scales)

A mixed-model ANOVA with total problems as the dependent variable revealed a significant main effect of group (*F*(1, 60) = 4.15, *p* =.046, *η*_p_^2^ = .07), but no significant main effect of time, nor a group by time interaction (*p*s > .05). LT children had more total behavior problems than did TLD children between the ages of 2 and 4. Next, a mixed-model repeated-measures MANOVA was tested with internalizing and externalizing behaviors as dependent variables; there was no significant main effect of group and time, nor a group by time interaction (*p*s > .05). A follow-up ANOVA with internalizing behaviors as the dependent variable revealed a main effect of time that approached significance (*F*(1, 60) = 5.14, *p* =.027, *η*_p_^2^ = .08); there was no significant main effect of group, nor a group by time interaction (*p*s > .05). A trend was present toward internalizing behaviors being greater at age 4 than at age 2.

#### Syndromes (subscales)

A mixed-model repeated-measures MANOVA was assessed with internalizing syndromes (emotionally reactive, anxious/depressed, somatic complaints, and withdrawn) as dependent variables. The model indicated an omnibus group by syndromes interaction that approached significance (Wilks’ lambda = 0.89, *F*(3, 58) = 2.30, *p* = .087, *η*_p_^2^ = .11), but no significant effect of group, nor a group by time interaction (*p*s > .05). A follow-up ANOVA with emotionally reactive dependent variable revealed a main effect of group that approached significance (*F*(1, 60) = 4.59, *p* =.036, *η*_p_^2^ = .07). LT toddlers exhibited a trend toward higher emotional reactivity than TLD toddlers between the ages of 2 and 4. The other follow-up ANOVA with anxious/depressed syndrome as the dependent variable revealed a significant main effect of time (*F*(1, 60) = 8.55, *p* =.005, *η*_p_^2^ = .13) and a main effect of group that approached significance (*F*(1, 60) = 4.92, *p* =.030, *η*_p_^2^ = .08). Anxiety/depression was higher at age 4 than at age 2, and LT toddlers exhibited a trend toward higher anxiety/depression than TLD toddlers between the ages of 2 and 4. Another mixed-model repeated-measures MANOVA was assessed with externalizing syndromes (attention and aggression) as dependent variables. No significant effects were found of group, nor group by time and group by syndrome interactions (*p*s > .05). Finally, a mixed-model ANOVA with sleep problems syndrome as the dependent variable revealed no significant effect of group, nor group by time and group by syndrome interactions (*p*s > .05).

Note that 33 items do not contribute to the seven syndrome subscales and are labeled “other problems” (e.g., “Afraid to try new things,” “Too shy or timid,” “Cries a lot,” and so forth) in the CBCL protocol. These items did not contribute to the internalizing and externalizing scale scores but only contributed to the total problems scale score. The LT children’s other problem scores at age 2 and at age 4 were 13.97 (*SD* = 5.89) and 12.81 (*SD* = 6.17), respectively. The TLD children’s other problem scores at age 2 and at age 4 were 10.29 (*SD* = 4.97) and 9.77 (*SD* = 5.12), respectively. A mixed-model ANOVA was used to assess how other problem raw scores changed from age 2 to 4 in the LT and TLD groups. A significant main effect of group (*F*(1, 60) = 8.49, *p* = .005, *η*_p_^2^ = .12) was found, but no main effect of time, nor a group by time interaction (*p*s > .05). For the other problems subscale, LT children exhibited greater behavioral problems than did TLD children between the ages of 2 and 4.

### Presence and absence of behavior problems: Fisher’s exact test

Table 4 lists the number of participants for all ten of the CBCL scales and subscales at both time points for the LT and TLD groups. Fisher’s exact test was used to test differences between the LT and TLD groups in the percentages of participants with behavior problems for each of the CBCL scales and subscales at ages 2 and 4. (1) The presence of behavior problems based on total problems (i.e., total problems scale) was greater in the LT group than in the TLD group at age 2 and at age 4 (*p*s =.037 and .024, respectively). (2) The difference between groups approached significance for internalizing behaviors at age 4 (*p* = .092). (3) For emotionally reactive and withdrawal behaviors, the difference between groups approached significance (*p*s =.052 and .056, respectively) at age 2. Moreover, the presence of behavior problems based on withdrawal behaviors was greater in the LT group than in the TLD group at 4 years of age, although the difference fell just outside significance (*p* = .013). Therefore, a trend toward a higher percentage of emotionally reactive behaviors was found in the LT group versus the TLD group at age 2. Furthermore, at both ages, there were higher percentages of the presence of total behavior problems (that is, the total problems scale) in the LT group than in the TLD group and a trend toward higher percentages of withdrawal behaviors in the LT group than in the TLD group.

**Table 4:** Presence of behavioral problems changed from time 1 (age 2) to time 2 (age 4) in the TLD and LT groups

	Presence at time 1			Presence at time 2			Presence at time 1 and time 2		Only presence at time 1		*p*^a^
	TLD	LT	*p*^a^	TLD	LT	*p*^a^	TLD	LT	TLD	LT	
Total problems	4	11	.037*	5	13	.024*	0	10	4	1	.004*
Internalizing problems	4	9	.106	8	14	.092+	3	7	1	2	.706
Externalizing problems	2	4	.336	2	3	.500	0	2	2	2	.400
Emotionally reactive	1	6	.052+	4	8	.335	0	3	1	3	.571
Anxious/depressed	0	3	.119	2	6	.255	0	1	0	2	1.000
Somatic complaints	1	3	.306	5	6	1.000	0	1	1	2	.750
Withdrawn	0	4	.056**+**	1	8	.013+	0	2	0	2	1.000
Attention	1	3	.306	1	0	1.000	0	0	1	3	1.000
Aggression	1	3	.306	2	1	1.000	0	1	1	2	.750
Sleep problems	3	5	.354	2	3	1.000	0	0	3	5	1.000

*TLD*, typical language development group (*n* = 31); *LT*, late-talking group (*n* = 31). Data were presented as *n*. *p* derived from Fisher’s exact test. ^a^Based on Bonferroni-corrected α level, *significant, +borderline significant

Table 4 also presents the percentage of consistent behavior problems in those children with behavior problems at age 2. Fisher’s exact tests showed that among LT and TLD toddlers with the presence of behavior problems at age 2, the LT group had a higher percentage of participants with stable behavior problems than did the TLD group in terms of total problems (*p* = .004). Overall, early behavior problems were more stable among LT children than among TLD children across very young ages. Furthermore, the percentage of the presence of withdrawal behaviors among LT children increased during toddlerhood and preschool age.

### Association between toddler vocabulary size and their behavior problems

Logistic regression revealed that toddlers with larger vocabularies were less likely to exhibit withdrawal behaviors at age 4 (odds ratio [OR] = 0.43, 95% confidence interval [CI] = [0.19, 0.95], *p* =.037) but not at age 2 (*p* >.05). Therefore, toddlers with large vocabularies were less likely to develop withdrawal behavior by age 4.

## Discussion

Unlike previous large-scale studies [3, 16, 17], this study used a rigorous matched pairs design following two groups of children from ages 2 to 4. This study had high retention rates, and the effect sizes were moderate to large. These factors increased the validity of the findings from this study [3, 16, 17]. Furthermore, this is one of the few longitudinal studies examining the temporal stability of behavior problems in LT toddlers across early childhood. The main findings are discussed below.

Regarding the early language delay being associated with developing behavior problems, according to parental reports, we found that LT children were developing more behavior problems than TLD children from a very young age. Moreover, LT children exhibit a trend to develop internalizing behaviors, such as emotional reactivity and withdrawal, across early years, but not externalizing behaviors. Previous findings indicated that LT children predominately exhibit internalizing behaviors in toddlerhood [15, 19] and at preschool ages [3, 16]. Additionally, other problem syndromes (e.g., afraid to try new things) only contributing to the total problems score were more severe in LT children than in TLD children during toddlerhood and preschool age. Therefore, in addition to focusing on the development of internalizing behaviors among LT toddlers, it is important to address other behavior problems that do not contribute to internalizing and externalizing problems.

Compared with TLD children, LT children tended to be more stable regarding the behavior problems. Previous studies reported only that LT toddlers have behavior problems at preschool ages [3, 16] and school ages [3, 17]. Nonetheless, our findings showed that there was persistence in the behavior problems of LT toddlers in the later developmental stage (e.g., preschool age) associated with the temporal stability of early behavior problems. In other words, LT children exhibit a trend of continuously experiencing more behavior problems than TLD children in emotional reactivity and anxious/depressed behaviors between toddlerhood and preschool age. Furthermore, among LT and TLD toddlers with the presence of behavior problems, the LT group had a higher percentage of participants with stable behavior problems than the TLD group in terms of total problems during preschool age. These results revealed the developmental continuity of early behavior problems through early childhood apparent from toddlerhood among LT children.

The association of language delay with temporal stability of behavior problems was found among LT children from a very young age. Three hypotheses may explain the nature of this association between language delay and behavior problems. For example, language delay and behavior problems may co-occur due to common biological (e.g., neurological injury or damage) or environmental risks (e.g., low parental education level or family income) that create broad developmental vulnerabilities [7, 36]. Alternatively, because language is a social behavior, language difficulties may impede positive interactions and lead to withdrawal and frustration [7]. Behavioral difficulties can interfere with language development by limiting the frequency and quality of interactions, thereby decreasing the exposure to rich linguistic input [36]. Understanding the processes underlying early language delays and persistent behavior problems in LT toddlers warrants further investigation with a larger sample size and a longitudinal design.

Both LT and TLD toddlers showed a common trend for increased internalizing behaviors, such as anxious and depressed behaviors, at age 4. One possible explanation is that parents had higher tolerance of their child’s behavior problems early in toddlerhood; however, they became more sensitive to such behavior problems during the preschool years [15]. Additionally, the percentage of children with increased social withdrawal behaviors was higher among LT toddlers than among TLD toddlers. One possible explanation is that daily learning activities and behavioral regulation demands exceed the language skills of LT children during early childhood. That is, parents may have noticed LT children’s behavior problems in their daily lives because LT children are inefficient at using language to regulate their behaviors. Thus, LT children feel frustrated and exhibit more internalizing behaviors. The mechanisms of change underlying these patterns of behavior problems among LT toddlers from toddlerhood to preschool age are complex and need further evaluation.

The sex ratio in this study was approximately 7:3 (male:female). This was uneven but similar to that of a previous report [17]. This may represent a natural sex distribution of LT toddlers in the community rather than a selection bias. A significant limitation of this study is that it employed the CBCL as the sole measure for categorizing behavior problems. Although the CBCL is an effective and inexpensive instrument for screening children’s behavior problems from a very young age, its results are applicable only to general behavior problems (e.g., aggression, somatic complaints, or emotional reactions). Other measures (i.e., behavioral observation in a standard experience context) could be used to assess the specific internalizing behaviors among LT toddlers. Furthermore, parents were the sole informants regarding their children’s behavior problems. Consequently, we could not assess the behavior problems exhibited by children outside the family context (e.g., in the classroom context) [37]. Future research should, therefore, also include teacher reports.

Finally, our findings imply that clinical professionals should assess the behavior problem profiles of LT toddlers when they are identified in toddlerhood and monitor the developmental patterns of these behaviors from that point. Moreover, toddlers with larger vocabularies were less likely to develop problematic withdrawal behavior in the preschool age. Early intervention is vital for these children due to their increased risk of maintaining and developing more severe behavior problems. Therefore, clinicians working with LT toddlers and their parents should focus not only on language skill promotion but also on behavior problem intervention.

## Conclusions

This 2-year prospective case-control study identified an association between early language delay with temporal stability of early behavior problems. A higher percentage of LT toddlers than TLD toddlers had persistent behavior problems. It is also important to note that toddlers with larger vocabularies were less likely to develop withdrawal behaviors over time. Early intervention is essential to decrease the likelihood of presenting behavior problems in later childhood years based on language delays. In addition, it is worth noting that delayed speech is often as frustrating for the child as it is for the parent who struggles to understand what the child is saying. We suggest that LT toddlers at risk of developing behavior problems should be identified and enrolled in early intervention services, which may also increase parents’ understanding of toddler communication and decrease their parenting stress.

## Data Availability

The datasets used or analyzed during the current study are available from the corresponding author on reasonable request.

## Abbreviations

LT: Late talking
TLD: Typical language development
